# RGS6 is an essential tumor suppressor that prevents bladder carcinogenesis by promoting p53 activation and DNMT1 downregulation

**DOI:** 10.18632/oncotarget.12473

**Published:** 2016-10-04

**Authors:** Jianqi Yang, Lance T. Platt, Biswanath Maity, Katelin E. Ahlers, Zili Luo, Zhibo Lin, Bandana Chakravarti, Stella-Rita Ibeawuchi, Ryan W. Askeland, Jolanta Bondaruk, Bogdan A. Czerniak, Rory A. Fisher

**Affiliations:** ^1^ Department of Pharmacology, Carver College of Medicine, University of Iowa, Iowa City, IA, USA; ^2^ Department of Pathology, Carver College of Medicine, University of Iowa, Iowa City, IA, USA; ^3^ Department of Pathology, MD Anderson Cancer Center, the University of Texas, Houston, TX, USA

**Keywords:** RGS6, urinary bladder cancer, tumor suppressor, p53, DNMT1

## Abstract

Urinary bladder cancer (UBC) is largely caused by exposure to toxic chemicals including those in cigarette smoke (i.e. BBN). An activating SNP in *RGS6* is associated with a pronounced reduction in UBC risk, especially among smokers. However, the mechanism underlying this reduction remains unknown. Here we demonstrate that RGS6 is robustly expressed in human urothelium, where urothelial cell carcinoma originates, and is downregulated in human UBC. Utilizing RGS6^−/−^ mice we interrogated a possible role for RGS6 as a tumor suppressor using the BBN-induced bladder carcinogenesis model that closely recapitulates human disease. As in humans, RGS6 is robustly expressed in mouse urothelium. RGS6 loss dramatically accelerates BBN-induced bladder carcinogenesis, with RGS6^−/−^ mice consistently displaying more advanced pathological lesions than RGS6^+/+^ mice. Furthermore, BBN treatment promotes urothelial RGS6 mRNA and protein downregulation. RGS6 loss impairs p53 activation and promotes aberrant accumulation of oncogenic protein DNMT1 in urothelium. Tumor suppressor RASSF1A, a DNMT1-regulated gene, is also silenced, likely *via* methylation of its promoter during BBN exposure. We hypothesize that this BBN-induced RGS6 loss represents a critical hit in UBC as it irrevocably impairs the anti-proliferative actions of the ATM/p53 and RASSF1A pathways. Consistent with these findings, RGS6^−/−^ mice treated with CP-31398, a p53-stablizing agent, and/or 5-Aza, a DNMT1 inhibitor, are protected from BBN-induced tumorigenesis. Together, our data identify RGS6 as a master tumor suppressor modulating two critical signaling pathways that are often dysregulated in UBC; therefore, RGS6 represents a potential novel biomarker for UBC diagnosis/prognosis and an appealing new target in its treatment.

## INTRODUCTION

Urinary bladder cancer (UBC), the fifth most common cancer in the U.S., is one of the deadliest malignancies worldwide [1]. In 2015 there were ~74,000 new cases of UBC and ~16,000 deaths caused by UBC in the U.S.. Due to its high recurrence and lifelong surveillance, UBC is the most costly cancer to treat on a per patient basis [2, 3]. Smoking is the primary risk factor for UBC, contributing to as many as two-thirds of all cases [4]. More than 90% of UBCs are urothelial cell carcinomas (also known as transitional cell carcinoma) [5], which can be classified into non-invasive and invasive subtypes, with the latter posing greater risk of metastases and lethality [6]. Studies in both human bladder tumor specimens and mouse models have implicated multiple signaling pathways in UBC carcinogenesis and progression [7]. First, mice lacking one allele of p53 [8] or that are deficient in both pRB/p53 [9] are highly susceptible to UBC induced by the carcinogen 4-Hydroxybutyl(butyl)nitrosamine (BBN), a chemical found in cigarette smoke. Second, hyperactivation of the H-Ras oncogene triggers bladder tumorigenesis [10] and aberrant upregulation of DNA methyltransferase 1 (DNMT1) is associated with increasing clinical stages of human UBC [11]. Finally, comprehensive molecular characterization of high-grade muscle-invasive urothelial bladder carcinomas in humans revealed that signaling through four pathways: 1) p53/Rb, 2) histone modification, 3) RTK/Ras/PI(3)K, and 4) SWI/SNF complex pathways, was altered *via* loss of tumor suppressor function or gain of oncogene function in tumors [12]. Despite this, our understanding of the pathogenic mechanisms underlying UBC initiation and progression remains insufficient and represents a critical barrier to UBC detection and treatment.

Regulator of G protein signaling 6 (RGS6) is a member of the RGS protein family, whose prototypic role is to negatively regulate heterotrimeric G protein signaling [13–17]. In addition, RGS6 also plays a critical role in cancer biology through G protein-independent mechanisms [18–21]. A SNP in the *RGS6* gene, which increases RGS6 expression, is associated with a significant reduction in the risk of human bladder cancer. In particular, this polymorphism in RGS6 was associated with a 34% reduction in bladder cancer incidence with stratified analyses revealing a 40% and 58% cancer reduction in smokers and in those who began smoking at young age, respectively [20]. However, the mechanism underlying this reduction in bladder cancer incidence is unknown. Recently, we showed that RGS6 loss 1) abolished doxorubicin-induced p53 activation by more than 90% in isolated cells and heart [22, 23] and 2) diminished DNMT1 degradation during Ras-induced transformation [18]. Given that both p53 loss and DNMT1 accumulation may promote bladder carcinogenesis [8, 11], we hypothesized that RGS6 functions as a master tumor suppressor in UBC by promoting both p53 activation and DNMT1 degradation. Using RGS6^−/−^ mice, we provide the first evidence that RGS6 loss accelerates BBN-induced UBC progression; and that p53 activation with CP-31398 [24], and/or DNMT1 inhibition with 5-Aza prevents tumor formation.

## RESULTS

**Figure 1:**
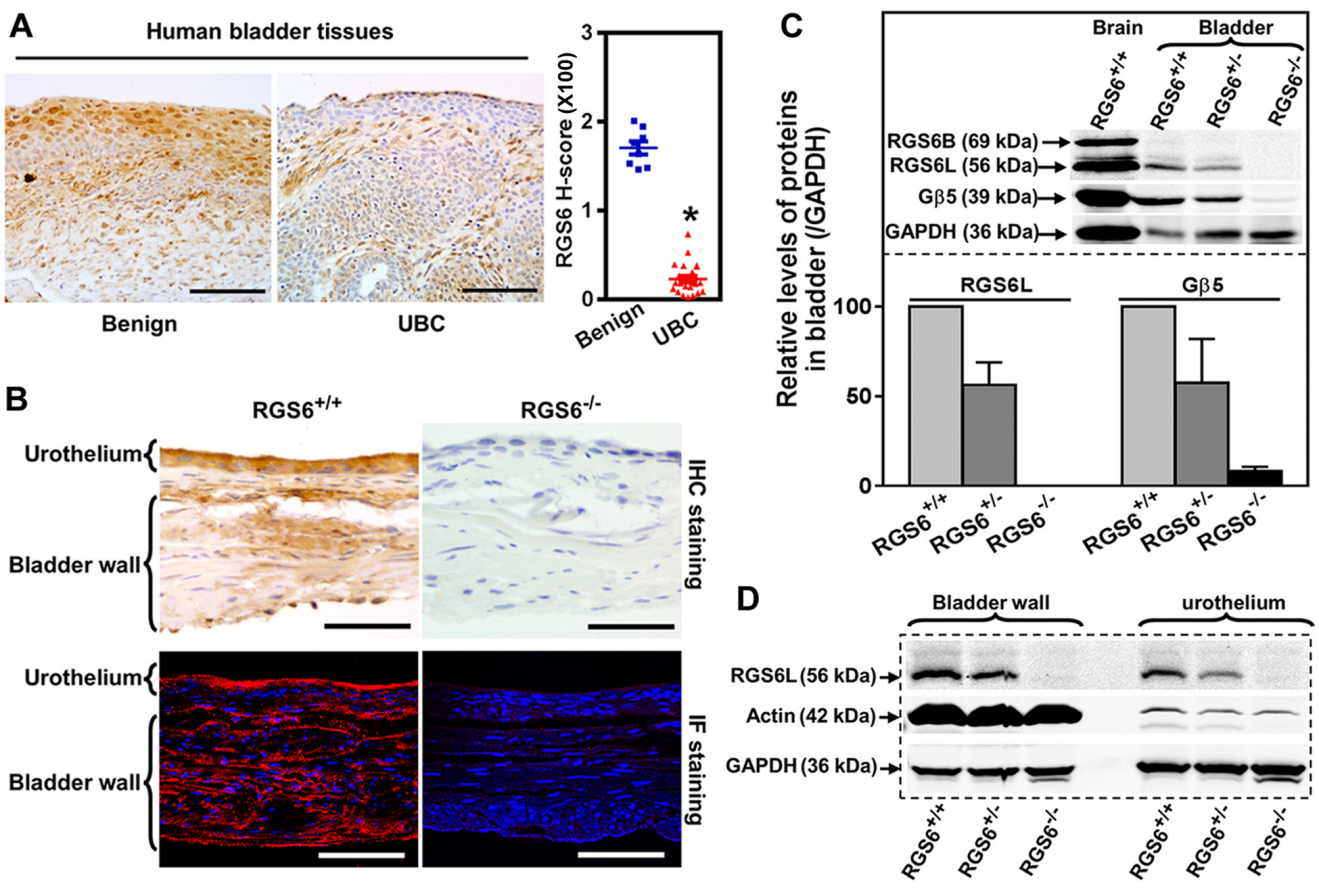
RGS6 is robustly expressed in human and mouse bladder and lost in human bladder tumors **A.** Expression of RGS6 in benign (*n* = 8) and UBC (*n* = 23) human bladder tissues. Scale bar, 100 μm. * *p* < 0.001. **B.** Detection of RGS6 in mouse bladder using immunohistochemical (IHC) and immunofluorescent (IF) staining. Scale bar, 50 μm. **C.** RGS6L is expressed in mouse bladder and stabilizes Gβ5. WB image are representative of three or more blots. Values of RGS6^+/+^ mice were arbitrarily set as 1. **D.** RGS6 was measured using WB in bladder wall and urothelium.

Given that an activating SNP in the human *RGS6* gene is associated with a reduced risk of bladder cancer [20], we examined the possibility that RGS6 functions as a tumor suppressor by examining its expression in UBC. Figures 1A and S1A show that while RGS6 is highly expressed within the urothelium of benign bladder, there is a marked loss of urothelial RGS6 expression, over 80% loss by H-score immunohistochemical analysis, in human UBC. This human patient data demonstrates that there is a reciprocal relationship between RGS6 expression and the presence/risk of UBC as might be expected if RGS6 functions as a tumor suppressor.

To determine whether RGS6^−/−^ mice could be used to interrogate the tumor suppressor role of RGS6 in bladder, we first characterized RGS6 expression in the mouse bladder. As in humans, RGS6 is highly expressed in mouse urothelium and is absent in bladders derived from RGS6^−/−^mice, as shown by immunohistochemical and immunofluorescence staining (Figure 1B). These results were corroborated by western blot (WB) analysis which revealed that RGS6 is expressed in bladders derived from RGS6^+/+^ mice but not RGS6^−/−^ mice and there is a gene dosage effect on RGS6 expression (Figure 1C). Consistent with the observed expression of RGS6 in mouse urothelium by immunostaining, when bladder urothelium was microscopically dissected and analyzed by immunoblotting we found strong expression of RGS6 in the urothelium of RGS6^+/+^ but not RGS6^−/−^ mice (Figure 1D). Successful dissection of the urothelium from the bladder muscle wall was confirmed by blotting for actin, whose expression is high in the muscle wall but low in the urothelium. Notably, we found that loss of RGS6 in bladder was associated with a comparable loss of Gβ5 (Figure 1C), which is degraded when it is not in complex with a member of the R7 subfamily of RGS proteins that include RGS6, RGS7, RGS9 and RGS11[25]. This finding suggested that RGS6 is the primary if not only member of the R7 subfamily in mouse bladder and that its loss is not compensated for by upregulation of other R7 RGS members. Indeed, aside from RGS6, all other R7 family members were undetectable in mouse bladders of all RGS6 genotypes (Figure S1B). These findings demonstrate that RGS6, a protein previously linked to reduced UBC risk, is highly expressed in the urothelium where approximately 90% of UBCs arise and that RGS6^−/−^ mice can be employed to study its tumor suppressor role in bladder.

**Figure 2:**
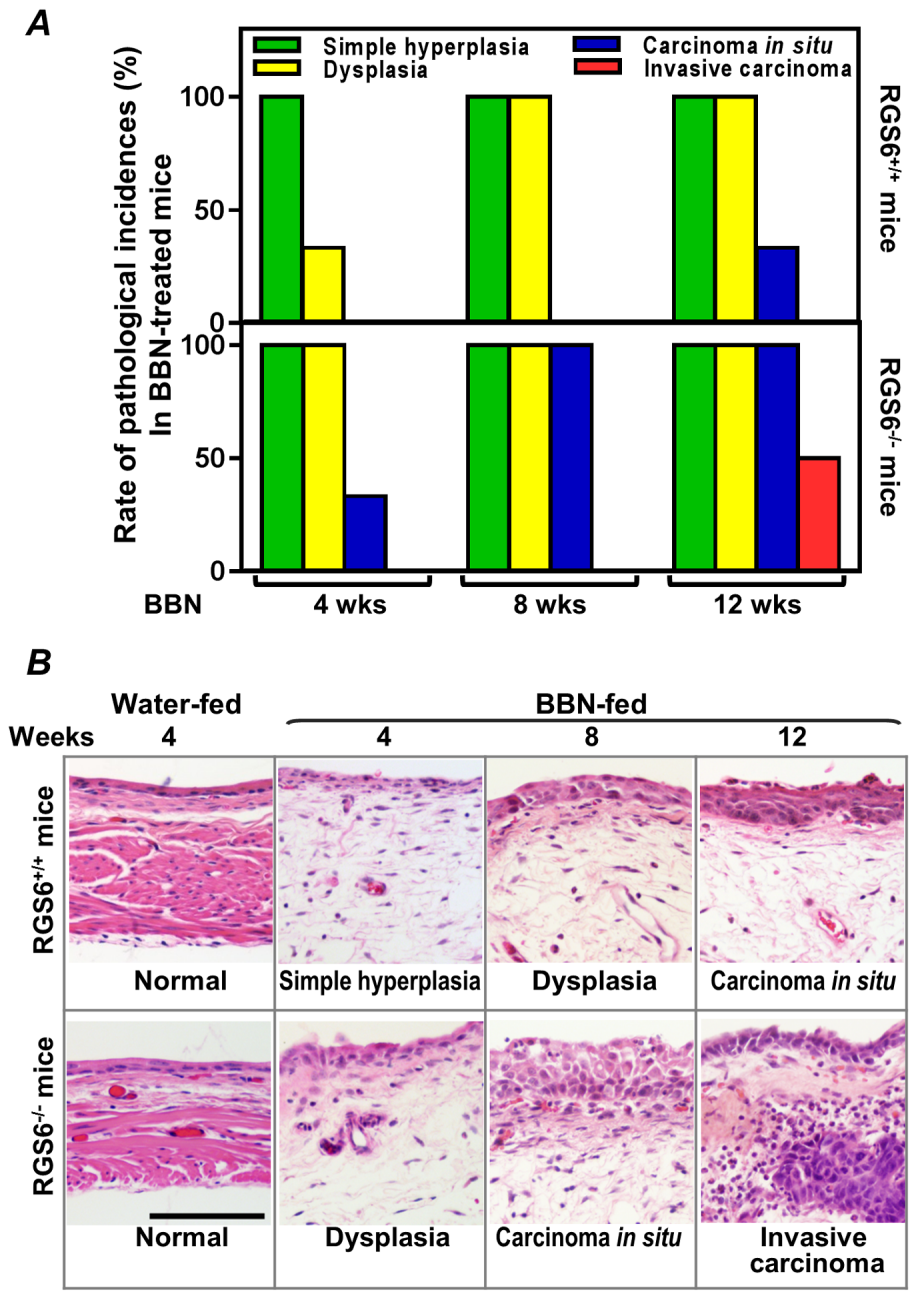
RGS6 inhibits BBN-induced bladder carcinogenesis **A.** Carcinogenesis was assessed by the pathological incidences (*n* = 3-6 mice/condition). **B.** Representative images of the most advanced pathological incidences at each time point. Scale bar, 100 μm.

**Figure 3:**
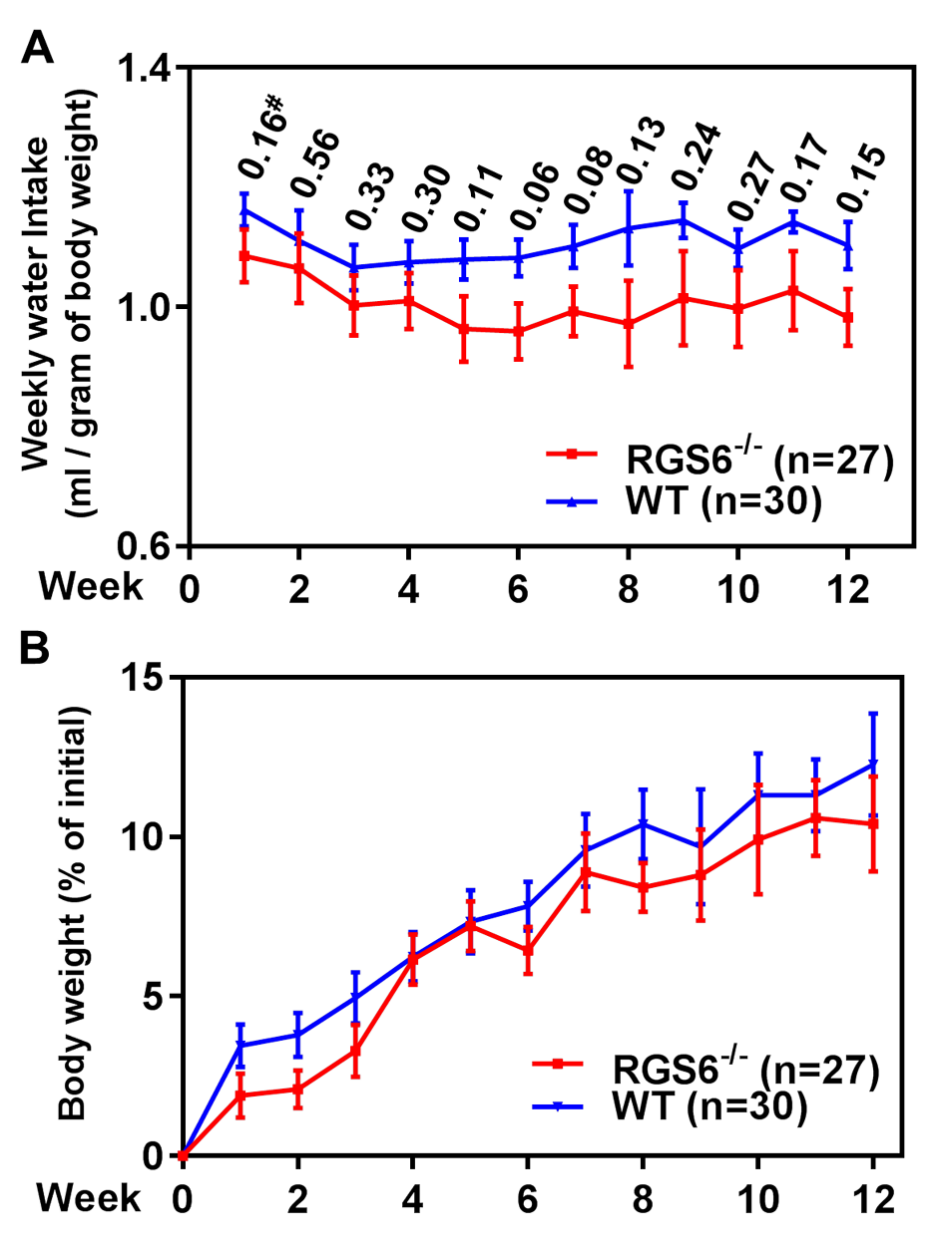
RGS6 loss does not alter BBN-water intake or body weight gain in mice RGS6^+/+^ and RGS6^−/−^ mice consumed a comparable amount of BBN-containing water **A.**, and had a similar rate of weight gain **B.** over 12 wks of BBN treatment. #, *p* values between RGS6^+/+^ and RGS6^−/−^ mice at each time point.

To interrogate the role(s) of RGS6 in the pathology of bladder carcinogenesis, we exposed mice to BBN, a carcinogen that induces UBC that has been shown to mimic human invasive bladder cancer[26]. RGS6^+/+^ and RGS6^−/−^ mice were given water or water containing 0.05% BBN for 4, 8, or 12 wks. As expected, no bladder tumors were detected in water-fed controls (not shown). However, in BBN-fed mice, RGS6 loss was associated with a significant acceleration in bladder cancer progression. This acceleration in bladder cancer progression can be seen in Figures 2A-2B, as RGS6^−/−^ mice consistently displayed more advanced pathological changes/lesions compared to RGS6^+/+^ mice at each time point analyzed. In particular, while 50% of BBN-treated RGS6^−/−^ mice had invasive tumors by 12 wks, no invasive tumors were detected in RGS6^+/+^ mice. In addition, while 100% of RGS6^−/−^ mice displayed carcinoma *in situ* by 12 wks of BBN treatment, only 33% of RGS6^+/+^ had lesions at that time (Figures 2A, S2). Additional cohorts of mice (in each cohort *n*= 3 -5 mice per time point per treatment), BBN induced the same pathological changes in bladders; and these bladders were collected for biochemical analyses. During BBN treatment, water intake and body-weight were monitored. RGS6^−/−^ mice consumed a similar, if not smaller, amount of BBN-containing water and had a similar weight gain as RGS6^+/+^ mice (Figure 3), excluding the possibility that observed accelerated carcinogenesis in RGS6^−/−^ mice was due to excess consumption of BBN. These findings provide the first *in vivo* evidence that RGS6 is a *bona fide* tumor suppressor whose loss accelerates bladder carcinogenesis.

**Figure 4:**
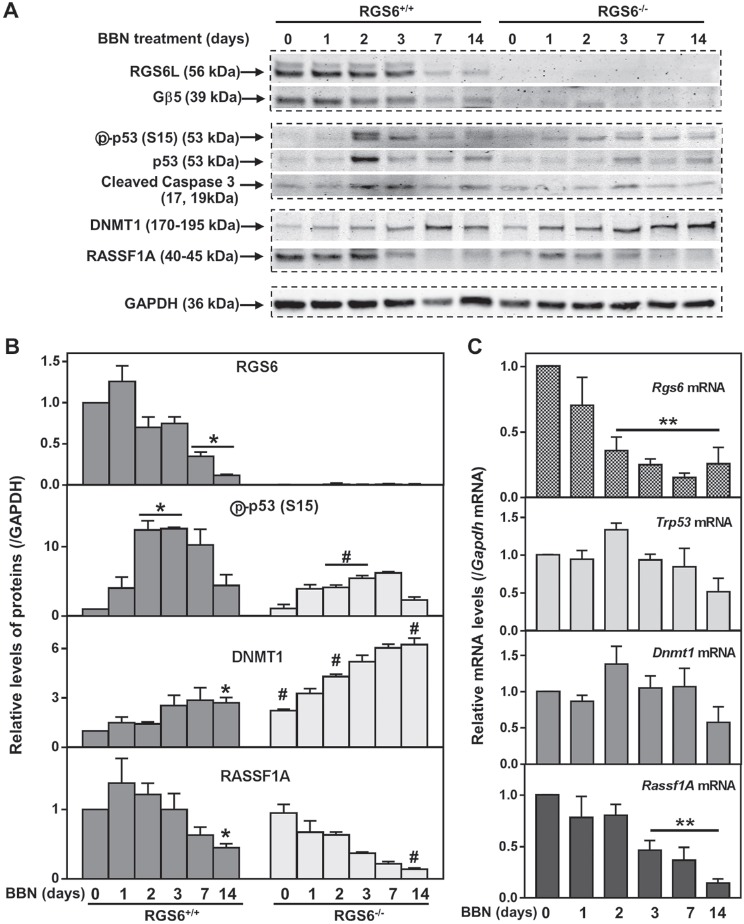
RGS6 loss exaggerates BBN-induced oncogenic signaling **A.** and **B.** Expression of key pro-oncogenic and -apoptotic proteins were measured *via* WB in the urothelium of mice given 0.05% BBN acutely for ≤14 days (*n* = 3 mice/time point). * *p* <0.03 vs day 0 of RGS6^+/+^; #, *p* <0.05, RGS6^−/−^ vs RGS6^+/+^. **C.** Relative mRNA levels of these proteins were determined using qRT-PCR in the bladder epithelium (*n* = 4 mice/time point). Values of day-0 RGS6^+/+^ mice were arbitrarily set as 1. **, *p* <0.05 vs day 0 of RGS6^+/+^.

To elucidate the molecular mechanism underlying the ability of RGS6 to suppress bladder carcinogenesis, RGS6^+/+^ mice were treated with 0.05% BBN acutely for 1, 2, 3, 7, 14 days or chronically for 12 wks. Following treatment, the animals were sacrificed and the urothelium microdissected for WB analysis of RGS6 expression. Figures 4A and 4B show that acute BBN treatment leads to a rapid and time-dependent downregulation of urothelial RGS6 and its binding partner Gβ5. By day 14 of BBN exposure, RGS6 protein expression was reduced by 88%, when compared to untreated RGS6^+/+^ mice. This downregulation of RGS6 mirrors a fast loss of urothelial RGS6 mRNA (Figure 4C). Interestingly, BBN exposure does not completely ablate urothelial RGS6 expression in RGS6^+/+^ mice, which could be the reason that tumor development is delayed for up to 8 wks in RGS6^+/+^ mice as compared to RGS6^−/−^ mice. Together, these findings demonstrate that BBN carcinogen exposure results in rapid RGS6 mRNA and protein loss within mouse urothelium.

Human bladder tumor progression is often associated with loss-of-function mutations in the tumor suppressor p53 [12]. Previously, we demonstrated that RGS6 is essential for doxorubicin- and DMBA-induced p53 activation as well as ROS generation in both isolated cells [21, 22] and in hearts from treated mice [23]. Similarly, we found that RGS6 is required for urothelial BBN-induced p53 activation and caspase 3 cleavage as the transient peak in p53 activation (phosphorylation of S15) and in caspase 3 cleavage in RGS6^+/+^ mice at 2-3 days following BBN exposure was significantly blunted in RGS6^−/−^ mice (Figures 4A-4B). In addition, RGS6 loss was associated with a reduction in ROS generation within the mouse bladder, similar to what we had seen in the heart (Figure S3). By 12wks of BBN treatment, phospho-p53 (S15) was undetectable in both RGS6^+/+^ and RGS6^−/−^ urothelium (not shown). Taken together, these data indicate that BBN-induced activation of p53 is RGS6-dependent. We hypothesize that it is this lack of p53 activation in RGS6^−/−^ mice following BBN treatment that is partially responsible for their vulnerability to carcinogen assault.

**Figure 5:**
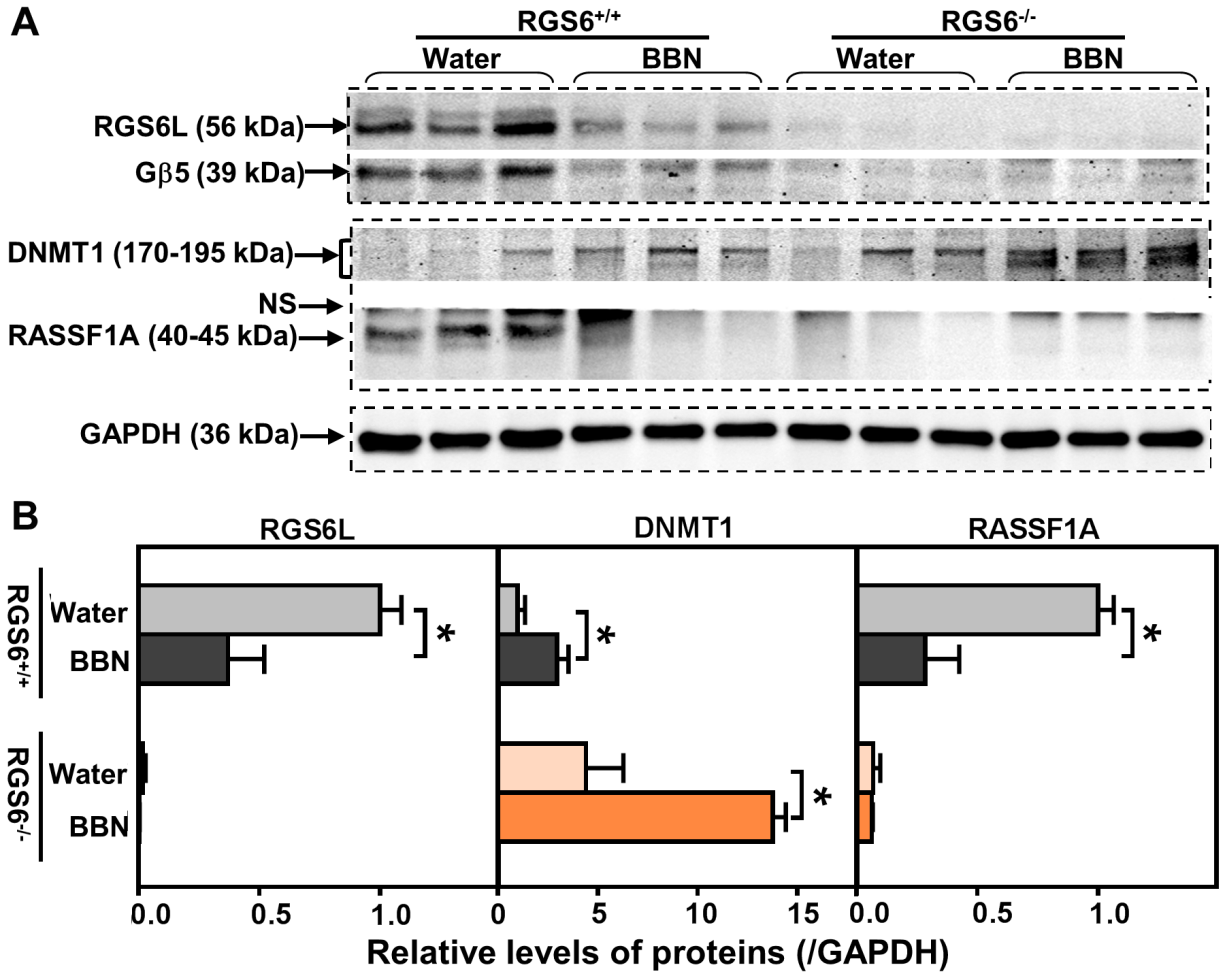
RGS6 loss exaggerated pro-oncogenic signaling in mice after chronic BBN exposure Mice were treated with 0.05% BBN water for 12 wks. Expression of RGS6L, Gβ5, DNMT1 and RASSF1A were measured using WB **A.** (*n* = 3 mice per treatment per genotype) and quantified in **B.** Values of water-fed RGS6^+/+^ mice were arbitrarily set as 1 * *p* < 0.05. NS, non-specific band.

p53 loss-of-function is only one signaling abnormality associated with human bladder tumor progression. Aberrant signaling *via* DNMT1 and its direct downstream target RASSF1A is also associated with bladder carcinogenesis [11, 27, 28]. We recently demonstrated that RGS6 facilitates Tip60-mediated DNMT1 degradation during Ras-induced cellular transformation [18]. Therefore, we measured urothelial DNMT1 and RASSF1A expression in BBN-treated RGS6^+/+^ and RGS6^−/−^ mice. BBN treatment was associated with a time-dependent increase in DNMT1 and a concomitant decrease in RASSF1A in RGS6^+/+^ urothelium (Figures 4A-4B). As expected, both of these changes in DNMT1 and RASSF1A expression were greatly exaggerated in RGS6^−/−^ mice (Figures 4A-4B). These changes were further exaggerated in animals chronically treated with BBN for 12 wks (Figure 5). We hypothesize that, together with the loss of p53 activation, the alteration in DNMT1 and RASSF1A levels, and presumably signaling, in BBN treated RGS6^−/−^ mice further contribute to their carcinogen vulnerability.

**Figure 6:**
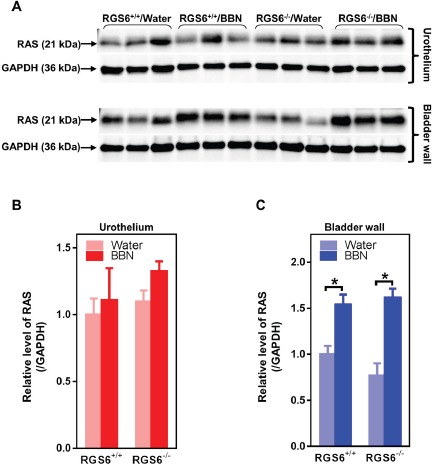
BBN treatment of mice has no effects on RAS expression in urothelium but induces RAS in bladder wall independent of RGS6. RGS6^+/+^ and RGS6^−/−^ mice were treated with 0.05% BBN for 12 wks Ras expression was determined in urothelium and bladder wall tissue lysates using western blot **A.** Quantification of RAS in urothelium and bladder wall is shown in **B.** and **C.** The values of RAS expression in tissue lysates from RGS6^+/+^ mice fed with water was arbitrarily set as 1.0 in B and C. * p < 0.02.

Concurrent mutations in the coding and regulatory regions of HRAS in urinary bladder tumors are associated with increased Ras expression [29], and constitutively active HRAS triggers bladder carcinogenesis in mouse urothelium [10]. Therefore, we measured RAS expression in the mouse bladder following 12 wks of BBN treatment in RGS6^+/+^ and RGS6^−/−^ mice. RAS levels remained unaltered in both RGS6^+/+^ and RGS6^−/−^ mouse urothelium. Instead, RAS expression was induced in the bladder wall in a RGS6-independent manner (Figures 6A-6C). Thus while BBN-induced increases in Ras expression in bladder wall may contribute to UBC development, the tumor suppressor actions of RGS6 are not involved in this signaling pathway.

**Figure 7:**
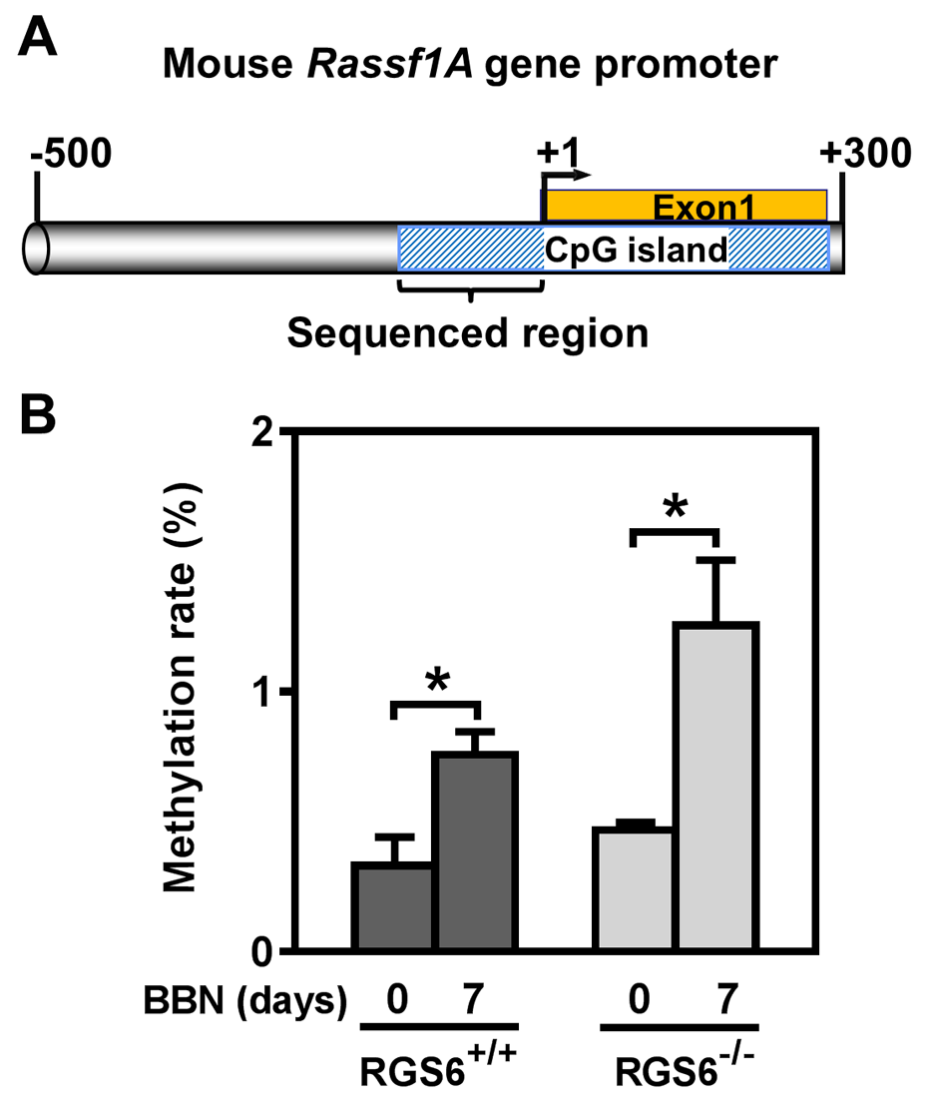
BBN induces methylation of a CpG island within the mouse *Rassf1A* gene promoter **A.** Schematic illustration of the CpG island in mouse *Rassf1A* gene promoter (drawing is in scale). **B.** Methylation rate (percentage of methylated CpG sites) of *Rassf1A* gene promoter was determined using bisulfite sequencing in mouse urothelium (*n* = 3 - 4), * *p* < 0.05.

**Figure 8:**
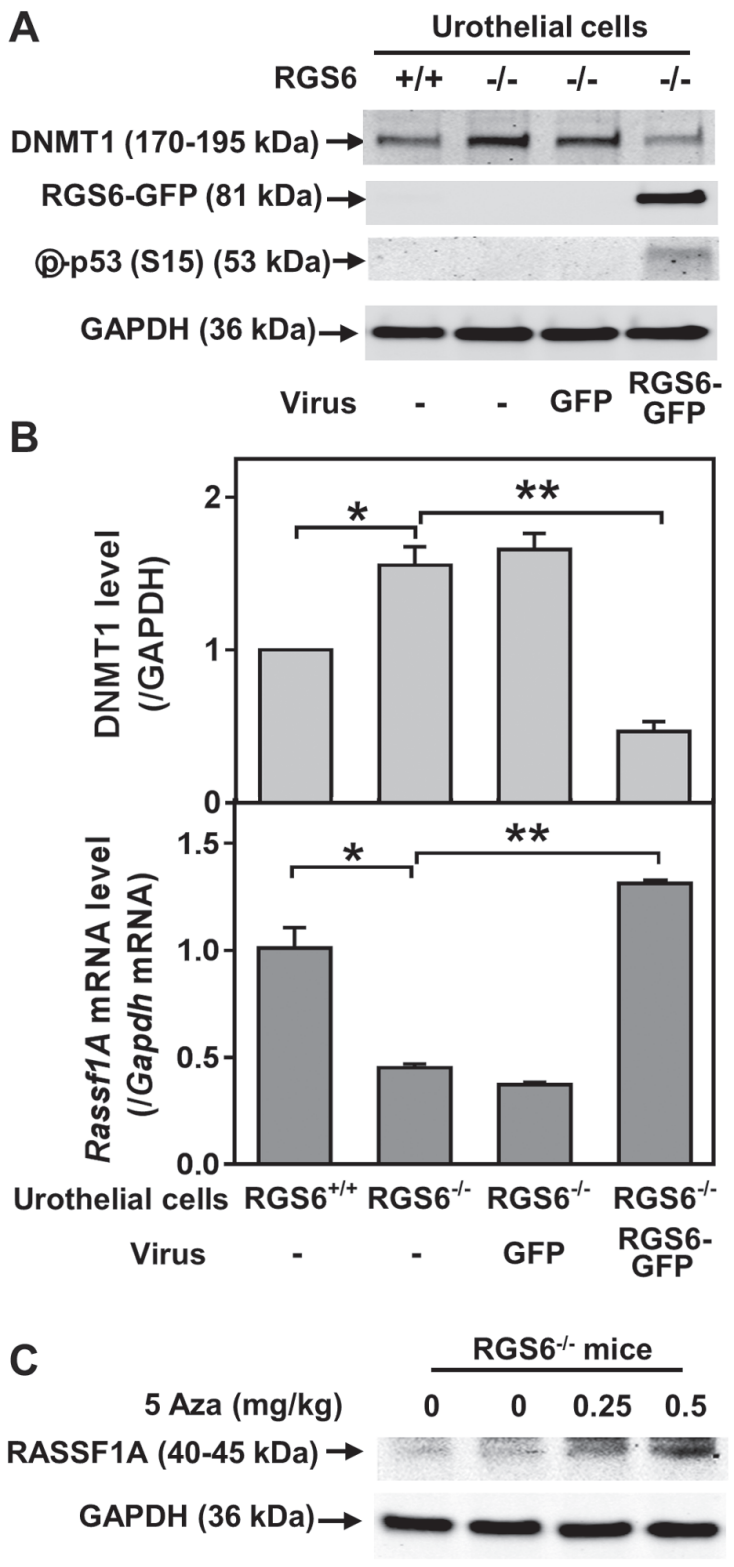
Expression of RGS6 in RGS6 urothelial cells reduces DNMT1 protein levels and increases *Rassf1A* mRNA level RGS6^+/+^ and RGS6^−/−^ mouse urothelial cell primary cultures were infected with or without (-) adenoviruses expressing GFP or RGS6Lα2-GFP. Levels of DNMT1 and phosphorylated p53 **A.** and *Rassf1A* mRNA **B.** were measured using WB and qRT-PCR, respectively (*n* = 3-4). *, *p* <0.005; **, *p* < 0.002. WB image are representative of three blots. **C.** 5-Aza treatment rescues RASSF1A expression in RGS6^−/−^ mice. Mice were treated with 5-Aza for two weeks. RASSF1A was measured *via* WB.

We further interrogated the link between RGS6 loss and the concurrent upregulation of DNMT1 and downregulation of RASSF1A. Based upon our prior finding that RGS6 promotes DNMT1 protein degradation [18] we hypothesized that loss of RGS6 during BBN induced carcinogenesis would promote stabilization of DNMT1 protein which would transcriptionally silence the tumor suppressor RASSF1A. Consistent with this idea, we found that DNMT1 mRNA levels were not significantly altered during BBN treatment while RASSF1A mRNA levels decreased in parallel with RASSF1A protein levels (Figures 4B-4C). As expected, we did not see a correlation between p53 induction, known to be controlled by phosphorylation induced protein stabilization [30], and its mRNA levels (Figures 4B-4C). Consistent with the increase in DNMT1 protein levels and loss of both RASSF1A proteins and mRNA, bisulfite analysis revealed a significant increase in the methylation status of the RASSF1A promoter in the bladder urothelium 7 days following BBN treatment of RGS6^+/+^ mice (Figure 7). Lastly, to test whether RGS6 loss is directly responsible for the upregulation of urothelial DNMT1 and in turn the silencing of RASSF1A expression, RGS6L was overexpressed in RGS6^−/−^ mouse bladder urothelial cell primary cultures. While RGS6 loss was associated with increases in DNMT1 protein expression and a repression of RASSF1A protein and mRNA level in urothelium of BBN-treated mice (Figure 4), overexpression of RGS6 (GFP-RGS6Lα2) completely reversed those changes (Figures 8A and 8B). Consistently, treating RGS6^−/−^ mice with the DNMT1 inhibitor 5-Aza also rescued RASSF1A expression (Figure 8C). Together, these findings demonstrate that RGS6 is essential for BBN-induced changes in urothelial DNMT1 and RASSF1A expression and that it directly modulates the DNMT1/RASSF1A pathway in urothelial cells.

Based on our findings, we hypothesize that acceleration of UBC development in RGS6^−/−^ mice was due to a combined action of insufficient activation of tumor suppressor p53 and aberrant accumulation of oncogenic protein DNMT1 in urothelium. To test this hypothesis, CP-31398 (p53-stablizing agent) and 5-Aza-2'-deoxycytidine (DNMT1 inhibitor) were administered to BBN-treated RGS6^−/−^ mice. Either drug alone or in combination protected RGS6^−/−^ mice from BBN-induced tumor formation (Table 1).

**Table 1:** Activation of p53 and/or inhibition of DNMT1 protects RGS6^−/−^ mice from BBN-induced bladder tumorigenesis

	Treatments			
BBN (0.05%, 12 wks)	+	+	+	+
5-Aza (inhibits DNMT1)	−	+	−	+
CP31398 (stabilizes / activates p53)	−	−	+	+
Bladder bearing solid tumor (# bladders examined)	6 (14)	1 (5)	0 (4)	0 (5)

BBN-fed RGS6^−/−^ mice were treated with CP-31398 and/or 5-Aza as described under Methods. At 12 wks of BBN treatment, mouse bladders were examined for solid tumors.

## DISCUSSION

**Figure 9:**
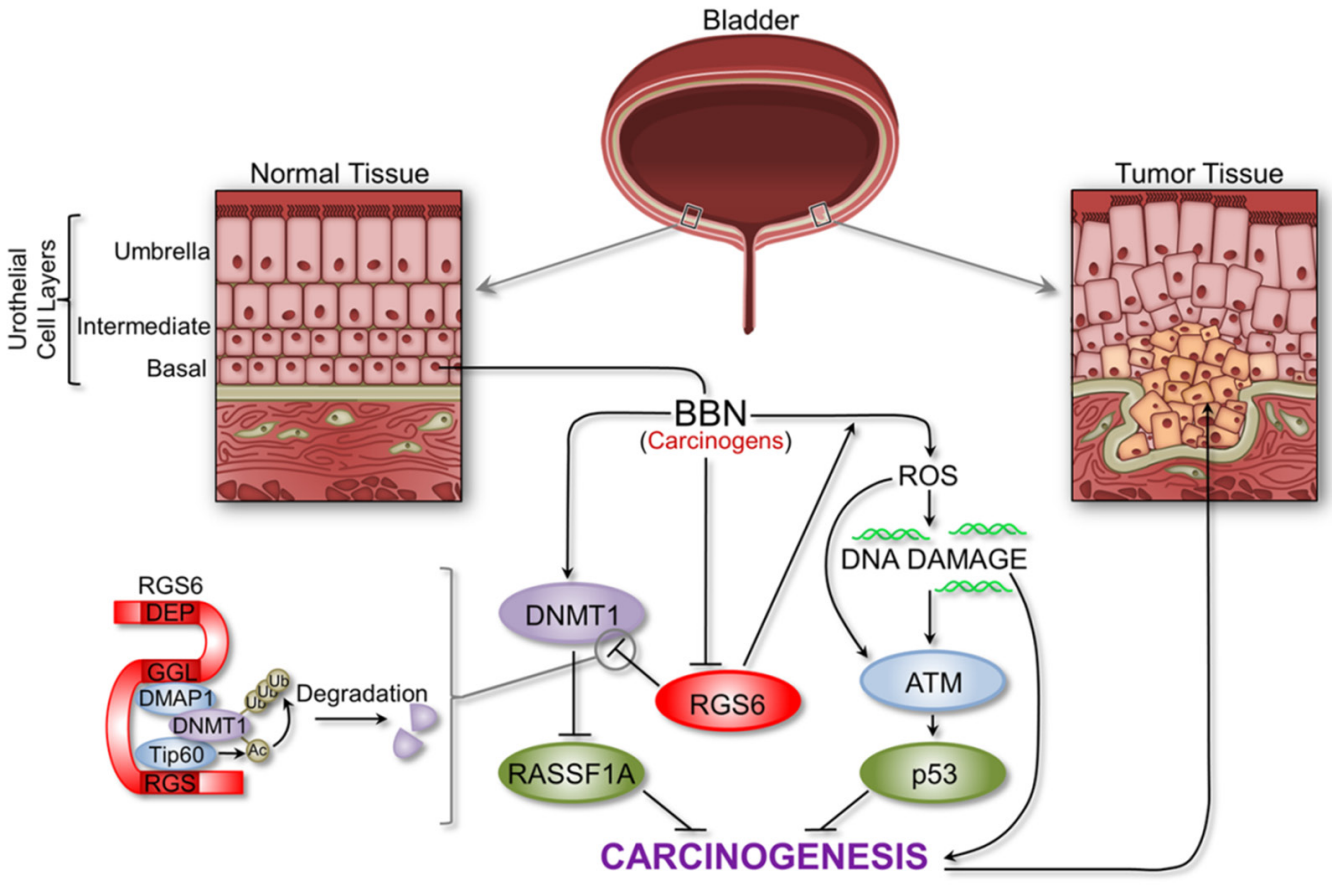
Schematic outlining the critical tumor suppressor role of RGS6 in bladder RGS6 loss impairs the ATM/p53 pathway while promoting increased DNMT1 expression and silencing of RASSF1A (see text for details). The proper regulation of these pathways is critical for the inhibition of bladder cancer development and progression.

This study identifies RGS6, a protein linked to reduced UBC risk among smokers, as a novel and critically important tumor suppressor in bladder using a smoke carcinogen model of UBC in mice that closely recapitulates human disease. We found that RGS6 is dramatically lost in human UBC and that loss of RGS6 in mice leads to downregulation of downstream tumor suppressors p53 and RASSF1A and upregulation of oncogenic DNMT1. Therefore we propose a model of carcinogen-induced bladder carcinogenesis in which RGS6 suppresses UBC by functioning as a critical upstream modulator of the ATM/p53 and DNMT1-RASSF1A signaling axes (Figure 9). We propose that RGS6 promotes both urothelial DNA repair *via* ATM/p53 activation and apoptosis following severe DNA damage in the early phase of carcinogen (BBN) exposure, thereby preventing genotoxic stress-induced oncogene activation. This is suggested by the loss of BBN-induced p53-S15 phosphorylation and p53 upregulation in RGS6^−/−^ vs RGS6^+/+^ bladders as well as our previous evidence that RGS6 is required for ATM activation and subsequent p53 induction and DNA repair induced by DMBA or doxorubicin [21, 22].

Our findings further show, however, that as BBN exposure of mice continues, urothelial RGS6 is downregulated transcriptionally. Downregulation of RGS6 not only impairs its function as a barrier to genotoxic stress, but leads directly to the accumulation of DNMT1 and silencing of the tumor suppressor RASSF1A, a known target for transcriptional silencing by DNMT1 [31, 32]. These findings are entirely consistent with our previous evidence that RGS6 functions as a scaffold protein to promote Tip60-mediated acetylation of DNMT1, followed by DNMT1 ubiquitylation and degradation[18]. In support of this latter mechanism, we showed that loss of RGS6 in bladders of BBN treated mice was associated with upregulation of DNMT1 by a transcriptionally-independent mechanism as well as transcriptional silencing of *Rassf1A* gene and methylation of its promoter. The fact that RGS6 is a key modulator of multiple signaling pathways associated with UBC initiation and progression [12, 33] further highlights the importance of this study as these synergistic effects have remained underappreciated.

These findings suggest a unique and multifarious role for RGS6 in bladder cancer pathogenesis. The striking loss of RGS6 in human UBC, together with our finding that an activating SNP in RGS6 is associated with a reduced risk of bladder cancer [20], strongly support the tumor suppressor role of RGS6 described here. Our studies in mice show that carcinogen-induced RGS6 loss in the bladder urothelium, where RGS6 is robustly expressed, appears to represent a crucial hit that drives carcinogenesis by impairing the anti-proliferative actions of p53 and RASSF1A. Consistent with a role of RGS6 as a critical upstream modulator of proteins that either suppress or promote (i.e. DNMT1) bladder carcinogenesis, we found that BBN-induced loss in urothelial RGS6 expression was temporally linked to alterations in expression of activated p53, RASSF1A and DNMT1. A recent molecular characterization of human invasive UBCs showed that p53 was impaired in 76% of bladder cancers [12]. The observed requirement for RGS6 in BBN-induced p53 activation in urothelium and the ability of the p53 activator CP-13398 to protect against bladder carcinogenesis due to RGS6 loss demonstrates the critical role of RGS6 in carcinogen-induced p53 activation in UBC. Further, though aberrant upregulation of DNMT1 has been linked to human invasive UBC [11], our findings provide critical new insights into the mechanisms underlying DNMT1 upregulation and its role in UBC. Our results are fully supportive of a key role for RGS6 loss in upregulation of DNMT1 in UBC and that DNMT1 is a key driver of UBC carcinogenesis as we found that 5-Aza prevents BBN-induced UBC in RGS6^−/−^ mice. In fact, RGS6 loss explains the aberrant expression of DNMT1 in UBC. The dramatic loss of urothelial RGS6 expression we found in human UBC would thus be expected to lead to dysregulation of p53 and the DNMT1/RASSF1A pathways, which our studies show independently contribute to accelerated UBC in RGS6^−/−^ mice.

The tumor suppressor actions of RGS6 described here are not likely due to alterations in Ras expression or signaling in the bladder urothelium. Though mutations in *HRAS* in urinary bladder tumors have been linked with increased Ras expression [29], their frequency is low (5% [12]). Moreover, over-expression of constitutively active HRAS in mouse urothelium induces superficial papillary tumors but not invasive UBC [10]. Here we found that BBN induces Ras expression in the bladder wall but not urothelium, where invasive UBC primarily originates, and that RGS6 loss did not alter Ras expression at either site. Therefore, BBN-induced upregulation of Ras in the bladder wall likely does not contribute to the increased incidence of bladder tumors in RGS6^−/−^ mice.

UBC is a deadly malignancy worldwide for which radical cystectomy and pelvic lymphadenectomy is the standard treatment for muscle invasive UBC. Though the Advanced Bladder Cancer Meta Analysis Collaboration showed that neoadjuvant platinum-based combination chemotherapy showed a significant benefit to survival and risk of death, the 5 yr overall survival increased only from 45% to 50% [34]. One of the barriers to better treatment of this disease remains insufficient understanding of the underlying mechanisms of UBC. Here we show a critical role of RGS6 in invasive UBC induced by a smoke carcinogen, responsible for the majority of UBC in humans. Given our evidence that RGS6 functions as a master tumor suppressor modulating two critical signaling pathways that are often dysregulated in UBC, RGS6 represents a potential novel biomarker for UBC diagnosis/prognosis as well as an appealing new target in its treatment. As such, it seems imperative that future work focus on identifying compounds that are capable of modulating RGS6 expression.

## MATERIALS AND METHODS

### Animals

We previously described the generation and genotyping of RGS6^−/−^ mice [35]. RGS6^−/−^ mice were backcrossed onto the C57BL6 background for five generations. Age-matched RGS6^+/+^ and RGS6^−/−^ mice were used for all experiments. All animal procedures were approved by the University of Iowa Institute for Animal Care and Use Committee.

### Treatment of mice

BBN (TCI America, B8061) was administered to mice *via* drinking water following the protocol described by Ohtani *et al.* [36]. Briefly, BBN was dissolved in tap water at a concentration of 0.05%. Mice were given BBN-containing water or tap water (controls) *ad libitum*. BBN-containing water was prepared every 2-3 days to achieve its maximal potency. Animal's body weight and water intake were recorded weekly. When needed, CP-31398 (A gift from Dr. Levy Kopelovich) was administered to mice daily *via* I.P. injection at a dose of 45 mg/kg for the first 7 days of BBN-treatment; 5-Aza-2'-deoxycytidine (5-Aza;Sigma, A3656) was administered to mice at a dose of 0.5 mg/kg three times per week (on Monday, Wednesday, and Friday) for the duration of BBN treatment.

### Isolation of mouse bladder urothelium

We developed a micro-dissection procedure to isolate bladder urothelium. Briefly, mouse bladders were cut into four pieces with sterile scissors and bladder pieces placed in one drop of 1 x PBS in a 60-mm tissue culture plate. Under the dissection microscope, the bladder muscle wall was separated from the urothelium at the edge of the tissue using two fine tweezers; the urothelium was then gently pulled away with one tweezer while the bladder muscle wall was held with another tweezer.

### Isolation and culture of mouse primary urothelial cells

Mouse primary urothelial cells were isolated from mouse bladder following a modified protocol described by Southgate *et al.* [37]. Briefly, mouse bladder urothelia were dissected and then incubated at 37°C for 2-4h in stripping solution (10 mM Hepes, 2 mM EDTA, 400 mg/L KCl, 60mg/L KH_2_PO_4_, 350 mg/L NaHCO_3_, 8000 mg/L NaCl, 48 mg/L Na_2_HPO_4_, and 1000 mg/L D-glucose). Following incubation, tissue was transferred to a new tube containing 0.1-0.5ml digestion solution (90 U/ml collagenase (Sigma, C5138), 0.2 mg/ml of DNase I, 10 mM Hepes, 140 mg/L CaCl_2_, 100 mg/L MgCl_2_-6H_2_O, 100 mg/L MgSO_4_-7H_2_O, 400 mg/L KCl, 60mg/L KH_2_PO_4_, 350 mg/L NaHCO_3_, 8000 mg/L NaCl, 48 mg/L Na_2_HPO_4_, and 1000 mg/L D-glucose) and was incubated at 37°C for 20 min with gentle shaking. To collect cells, digestion solution was mixed with 0.4 ml of culture medium (DMEM/F12 - 1:1;Invitrogen, 11320) supplemented with 100 U/ml of penicillin, 100 μg/ml of Streptomycin, and 10% FBS and centrifuged at 300 x g for 1 min. Urothelial cell pellets were then re-suspended in culture medium for plating. To promote urothelial cell attachment the culture dish was coated with type IV collagen (Sigma, C7521) at a density of 0.5 μg/cm^2^ three hours prior to the isolation. Prior to plating, urothelial cells were mixed with Ad-GFP or Ad-GFP-RGS6Lα2.

### H&E, immunohistochemical, and immuno-fluorescence staining

Female RGS6^+/+^ and RGS6^−/−^ mice were treated with 0.05% BBN water for 4, 8, and 12 wks. After animals were sacrificed, their bladders were rinsed with 0.1-0.2 ml of cold 1x PBS *via* a catheter inserted into the bladder transurethrally, and then inflated by 0.1 ml of 4% PFA *via* the same catheter. The neck of the bladder was ligated to keep PFA fixative inside. After fixation, bladders were processed at the University of Iowa Central Microscopy core. Each bladder was cut in two halves sagittally, embedded in paraffin, and stained with hematoxylin and eosin (H&E staining) for examining pathological changes/lesions. Some paraffin sections were used for immunohistochemical (IHC) staining using antibodies specific for RGS6 (home-made). RGS6 expression level was determined by immunohistochemical staining as we previously described [21]. A histo-score (H-score) was calculated by multiplying the percentage of positive cells by the average intensity. Additionally, bladders from three RGS6^+/+^ and three RGS6^−/−^ mice were also fixed as described above and embedded in optimum cutting temperature (OCT) medium for preparation of frozen sections to be used for immunofluorescence staining of RGS6. Formalin-fixed, paraffin-embedded human bladder sections were obtained from both the University of Iowa Department of Pathology and MD Anderson Cancer Center.

### Immunoblotting

Mouse tissues/cells were homogenized in ice-cold RIPA buffer (Santa Cruz, sc-24948) freshly supplemented with 1x protease inhibitor (Roche, 77836170001) and 1x phosphatase inhibitor (Sigma, P5726). Homogenates were centrifuged at 12,000 x g for 10 min at 4°C, and resulting supernatants were used for protein analyses. Western blot analysis was performed as previously described [22]. Briefly, 5-20 μg of protein were subjected to SDS PAGE electrophoresis and transferred to a nitrocellulose membrane. The resultant membrane was then incubated in blocking buffer (5% nonfat dried milk in 24.8 mM Tris, 137 mM NaCl, 2.7 mM KCl, and 0.1% Tween 20, pH 7.4) for 1h at room temperature, in primary antibody solution (diluted in blocking buffer) overnight at 4°C, and in secondary antibody solution (diluted in blocking buffer) for 1 h at room temperature. For phosphorylated protein immunoblotting, 5% nonfat milk in the blocking buffer was replaced with 3% BSA. Secondary antibodies were labeled with IRDye 800CW (Li-Cor Biosciences, 926-3211). Immunoreactive proteins were detected with the Odyssey infrared imaging system (Li-Cor Biosciences). Primary antibodies used for blotting were as follows: RGS6 (anti-RGS6L, home-made [23], 1:1000 dilution), Gβ5 (a gift from Dr. William F Simonds, 1:1000 dilution), RGS7 (a gift from Dr Vladlen Z Slepak, 1:2000 dilution), RGS9-1 (a gift from Dr Jason Chen, 1:4000 dilution), RGS11 (a gift from Dr Jason Chen, 1:1000 dilution), GAPDH (Sigma, G9545, 1:1000 dilution), Actin (Sigma A2066, 1:5000 dilution), p53 (Millipore CBL404, 1:700 dilution), phosphorylated p53(S15) (Cell signaling 9284S, 1:2000 dilution), DNMT1 (Santa Cruz, sc-20701, 1:1000 dilution), RASSF1A (eBioscience, 14-6888, 1:500 dilution), and α-tubulin (CalBiochem, CP06-100UG, 1:5000 dilution).

### Quantitive real-time PCR

Levels of mRNA were measured as we described previously [35]. Briefly, total RNA was extracted from mouse tissues or mouse primary urothelial cells infected with virus for 4 days, using a Qiagen RNeasy kit (Qiagen, 74104). First strand cDNA was synthesized from 100 ng of total RNA using SuperScriptIII First Strand Synthesis system (Invitrogen, 18080051). Real-Time PCR was carried out using iQ™ SYBR^®^ Green Supermix (Bio-Rad, 1708880), and the manufacturer's protocol. The following primers were used:

**Table:** 

Rgs6 forward	ATG GAG GGA GAT ACA CAT TTG AAG ATG CC
Rgs6 reverse	CAG CGA CTT TCC CTT CTT CTT GGC C
Trp53 forward	CCT TCT CAA AAA ACT TAC CAG GGC AAC TAT G
Trp53 reverse	GGG GAG GAG AGT ACG TGC ACA TAA CAG
Dnmt1 forward	GAG GAG AGA GAC CAG GAT AAG AAA CGC AGA
Dnmt1 reverse	TTG TCA TCT TCC TGT TCA CAT GGC TCT TCC
Rassf1A forward	TGC GAC CTC TGT GGA GAC TTC ATC
Rassf1A reverse	ACA GGA CGC ACT AGT TTC AGC TGA
Gapdh forward	GCA TGG CCT TCC GTG TTC CTA C
Gapdh reverse	GAT GCC TGC TTC ACC ACC TTC TTG

### Bisulfite sequencing

Genomic DNA was extracted from mouse urothelium and processed for bisulfite conversion using Zymo research EZ DNA methylation-lightning Kit (Zymo Research Corp, D5031), using the manufacturer's protocol. *Rassf1A* gene promoter was amplified from the bisulfite-converted genomic DNA using two primers: Methy-Rassf1A forward, GAA GGG GTT TTT TGA AAG GGT TTA TTT TTG TGT; Methy-Rassf1A reverse, CTC AAA CAC TCT CCC TAA TAA AAC AAA ACC AAA AAA TC. Resultant PCR products were analyzed with 1.0% DNA-agarose gels, purified using QIAquick gel extraction kit (Qiagen, 28704), and then subcloned into pCRII TOPO vector, using TOPO TA cloning kit (Invitrogen, 451641). For each urothelium, 9-15 colonies were picked, plasmids were extracted and sequenced at the University of Iowa Genomics Division. The sequence of each plasmid DNA was then aligned to WT *Rassf1A* gene promoter sequence. Methylation rate was calculated as a percentage of total number of methylated CpG sites over total CpG sites in the sequenced promoter region.

### Statistics

Significance was calculated using an unpaired Student's t-test. When multiple time points were assessed, a Bonferroni adjustment for multiple hypotheses was applied. *p* ≤ 0.05 was considered statistically significant. Data was expressed as means ± SEM (standard error of the mean).

## SUPPLEMENTARY MATERIAL



## References

[1] Knowles MA, Hurst CD (2015). Molecular biology of bladder cancer: new insights into pathogenesis and clinical diversity. Nat Rev Cancer.

[2] Kobayashi T, Owczarek TB, McKiernan JM, Abate-Shen C (2015). Modelling bladder cancer in mice: opportunities and challenges. Nat Rev Cancer.

[3] Mitra N, Indurkhya A (2005). A propensity score approach to estimating the cost-effectiveness of medical therapies from observational data. Health Econ.

[4] Brennan P, Bogillot O, Cordier S, Greiser E, Schill W, Vineis P, Lopez-Abente G, Tzonou A, Chang-Claude J, Bolm-Audorff U, Jockel KH, Donato F, Serra C (2000). Cigarette smoking and bladder cancer in men: a pooled analysis of 11 case-control studies. Int J Cancer.

[5] Kaufman DS, Shipley WU, Feldman AS (2009). Bladder cancer. Lancet.

[6] Gui Y, Guo G, Huang Y, Hu X, Tang A, Gao S, Wu R, Chen C, Li X, Zhou L, He M, Li Z, Sun X (2011). Frequent mutations of chromatin remodeling genes in transitional cell carcinoma of the bladder. Nature genetics.

[7] Thomas CY, Theodorescu D (2006). Molecular markers of prognosis and novel therapeutic strategies for urothelial cell carcinomas. World journal of urology.

[8] Ozaki K, Sukata T, Yamamoto S, Uwagawa S, Seki T, Kawasaki H, Yoshitake A, Wanibuchi H, Koide A, Mori Y, Fukushima S (1998). High susceptibility of p53(+/-) knockout mice in N-butyl-N-(4-hydroxybutyl)nitrosamine urinary bladder carcinogenesis and lack of frequent mutation in residual allele. Cancer Res.

[9] He F, Mo L, Zheng XY, Hu C, Lepor H, Lee EY, Sun TT, Wu XR (2009). Deficiency of pRb family proteins and p53 in invasive urothelial tumorigenesis. Cancer Research.

[10] Mo L, Zheng X, Huang HY, Shapiro E, Lepor H, Cordon-Cardo C, Sun TT, Wu XR (2007). Hyperactivation of Ha-ras oncogene, but not Ink4a/Arf deficiency, triggers bladder tumorigenesis. The Journal of clinical investigation.

[11] Wu CT, Wu CF, Lu CH, Lin CC, Chen WC, Lin PY, Chen MF (2011). Expression and function role of DNA methyltransferase 1 in human bladder cancer. Cancer.

[12] Network TCGAR (2014). Comprehensive molecular characterization of urothelial bladder carcinoma. Nature.

[13] Berman DM, Kozasa T, Gilman AG (1996). The Gtpase-Activating Protein Rgs4 Stabilizes the Transition State For Nucleotide Hydrolysis. Journal of Biological Chemistry.

[14] Dohlman HG, Thorner J (1997). RGS proteins and signaling by heterotrimeric G proteins. J Biol Chem.

[15] De Vries L, Zheng B, Fischer T, Elenko E, Farquhar MG (2000). The regulator of G protein signaling family [Review]. Annual Review of Pharmacology & Toxicology.

[16] Ross EM, Wilkie TM (2000). GTPase-activating proteins for heterotrimeric G proteins: Regulators of G protein signaling (RGS) and RGS-like proteins [Review]. Annual Review of Biochemistry.

[17] Chatterjee TK, Liu Z, Fisher RA (2003). Human RGS6 Gene Structure, Complex Alternative Splicing, and Role of N Terminus and G Protein gamma-Subunit-like (GGL) Domain in Subcellular Localization of RGS6 Splice Variants. J Biol Chem.

[18] Huang J, Stewart A, Maity B, Hagen J, Fagan RL, Yang J, Quelle DE, Brenner C, Fisher RA (2014). RGS6 suppresses Ras-induced cellular transformation by facilitating Tip60-mediated Dnmt1 degradation and promoting apoptosis. Oncogene.

[19] Luo Y, Qin SL, Yu MH, Mu YF, Wang ZS, Zhong M (2015). Prognostic value of regulator of G-protein signaling 6 in colorectal cancer. Biomed Pharmacother.

[20] Berman DM, Wang Y, Liu Z, Dong Q, Burke LA, Liotta LA, Fisher R, Wu X (2004). A functional polymorphism in RGS6 modulates the risk of bladder cancer. Cancer Res.

[21] Maity B, Stewart A, O'Malley Y, Askeland RW, Sugg SL, Fisher RA (2013). Regulator of G protein signaling 6 is a novel suppressor of breast tumor initiation and progression. Carcinogenesis.

[22] Huang J, Yang J, Maity B, Mayuzumi D, Fisher RA (2011). Regulator of G protein signaling 6 mediates doxorubicin-induced ATM and p53 activation by a reactive oxygen species-dependent mechanism. Cancer Research.

[23] Yang J, Maity B, Huang J, Gao Z, Stewart A, Weiss RM, Anderson ME, Fisher RA (2013). G-protein inactivator RGS6 mediates myocardial cell apoptosis and cardiomyopathy caused by doxorubicin. Cancer Res.

[24] Demma MJ, Wong S, Maxwell E, Dasmahapatra B (2004). CP-31398 restores DNA-binding activity to mutant p53 *in vitro* but does not affect p53 homologs p63 and p73. The Journal of Biological Chemistry.

[25] Chen CK, Eversole-Cire P, Zhang H, Mancino V, Chen YJ, He W, Wensel TG, Simon MI (2003). Instability of GGL domain-containing RGS proteins in mice lacking the G protein beta-subunit Gbeta5. Proc Natl Acad Sci U S A.

[26] Williams PD, Lee JK, Theodorescu D (2008). Molecular credentialing of rodent bladder carcinogenesis models. Neoplasia.

[27] Chan MW, Chan LW, Tang NL, Lo KW, Tong JH, Chan AW, Cheung HY, Wong WS, Chan PS, Lai FM, To KF (2003). Frequent hypermethylation of promoter region of RASSF1A in tumor tissues and voided urine of urinary bladder cancer patients. International journal of cancer Journal international du cancer.

[28] Lee MG, Kim HY, Byun DS, Lee SJ, Lee CH, Kim JI, Chang SG, Chi SG (2001). Frequent epigenetic inactivation of RASSF1A in human bladder carcinoma. Cancer Research.

[29] Czerniak B, Cohen GL, Etkind P, Deitch D, Simmons H, Herz F, Koss LG (1992). Concurrent mutations of coding and regulatory sequences of the Ha-ras gene in urinary bladder carcinomas. Hum Pathol.

[30] Ashcroft M, Kubbutat MH, Vousden KH (1999). Regulation of p53 function and stability by phosphorylation. Mol Cell Biol.

[31] Qiu X, Zhang L, Lu S, Song Y, Lao Y, Hu J, Fan H (2014). Upregulation of DNMT1 mediated by HBx suppresses RASSF1A expression independent of DNA methylation. Oncology reports.

[32] Agarwal S, Amin KS, Jagadeesh S, Baishay G, Rao PG, Barua NC, Bhattacharya S, Banerjee PP (2013). Mahanine restores RASSF1A expression by down-regulating DNMT1 and DNMT3B in prostate cancer cells. Molecular cancer.

[33] Zhou H, Huang HY, Shapiro E, Lepor H, Huang WC, Mohammadi M, Mohr I, Tang MS, Huang C, Wu XR (2012). Urothelial tumor initiation requires deregulation of multiple signaling pathways: implications in target-based therapies. Carcinogenesis.

[34] Advanced Bladder Cancer Meta-analysis C (2005). Neoadjuvant chemotherapy in invasive bladder cancer: update of a systematic review and meta-analysis of individual patient data advanced bladder cancer (ABC) meta-analysis collaboration. Eur Urol.

[35] Yang J, Huang J, Maity B, Gao Z, Lorca RA, Gudmundsson H, Li J, Stewart A, Swaminathan PD, Ibeawuchi SR, Shepherd A, Chen CK, Kutschke W (2010). RGS6, a modulator of parasympathetic activation in heart. Circulation Research.

[36] Ohtani M, Kakizoe T, Nishio Y, Sato S, Sugimura T, Fukushima S, Niijima T (1986). Sequential changes of mouse bladder epithelium during induction of invasive carcinomas by N-butyl-N-(4-hydroxybutyl)nitrosamine. Cancer Res.

[37] Southgate J, Masters JRW, Trejdosiewicz LK (2002). Culture of Human Urothelium. Culture of Epithelial Cells.

