# Supplementary material for: RGS6 is an essential tumor suppressor that prevents bladder carcinogenesis by promoting p53 activation and DNMT1 downregulation
Source: Oncotarget. 2016 Oct 4;7(43):69159–72. doi: 10.18632/oncotarget.12473 (PMC5342467; doi:10.18632/oncotarget.12473)
Supplement: Supplementary file 1 [file oncotarget-07-69159-s001.pdf]

RGS6 is an essential tumor suppressor that prevents bladder carcinogenesis by promoting p53 activation and DNMT1 downregulation

Supplementary Material

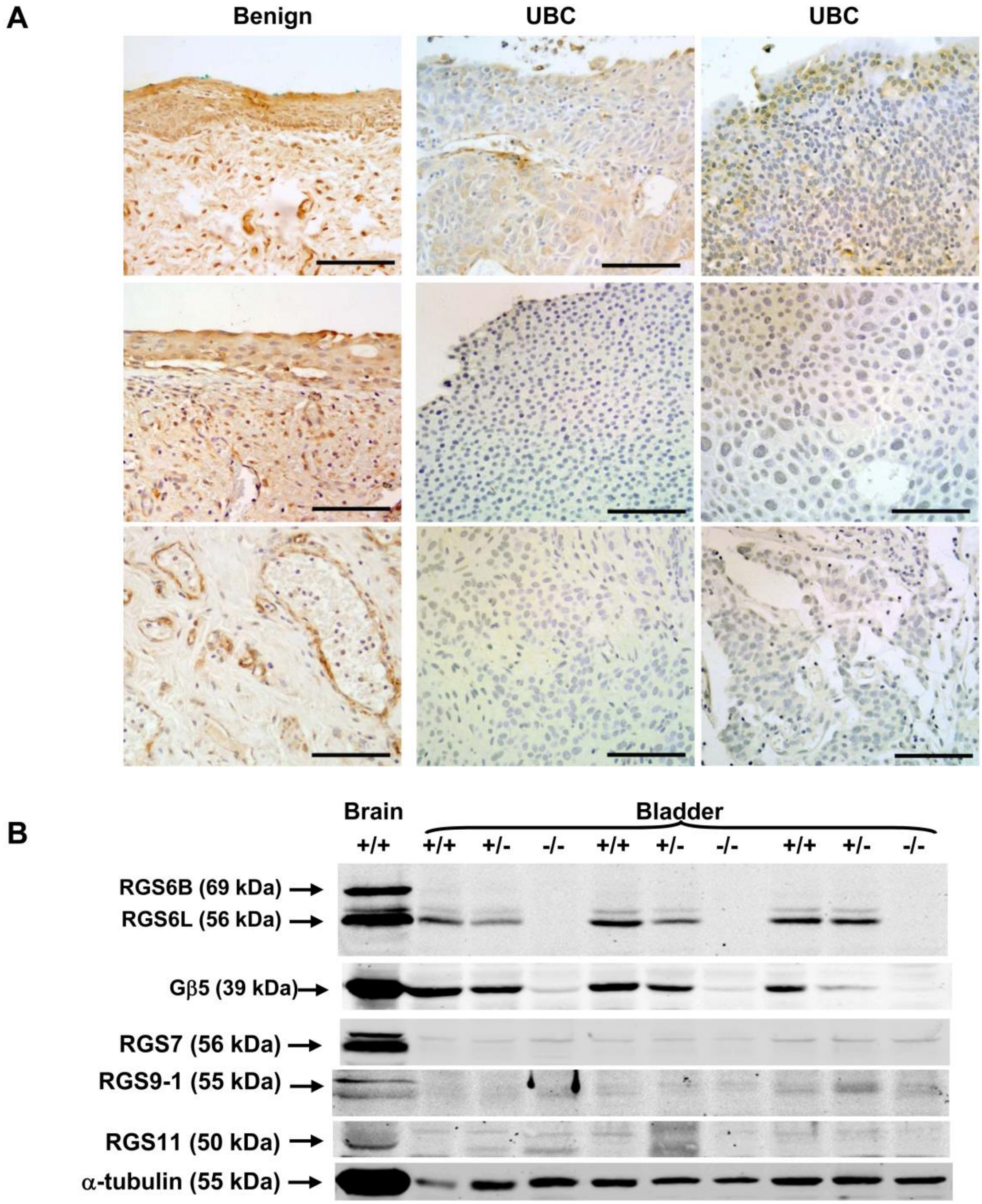

Fig S1. (**A**) Additional images showing dramatic loss of RGS6 (IHC staining, brown) in UBC human bladder tissue. Scale bar, 100 μm (**B**) RGS6 is the primary R7 subfamily RGS protein expressed in mouse bladder. Levels of RGS6, RGS7, RGS9-1, RGS11, and α-tubulin were determined in 20 μg of bladder lysates using western blot.

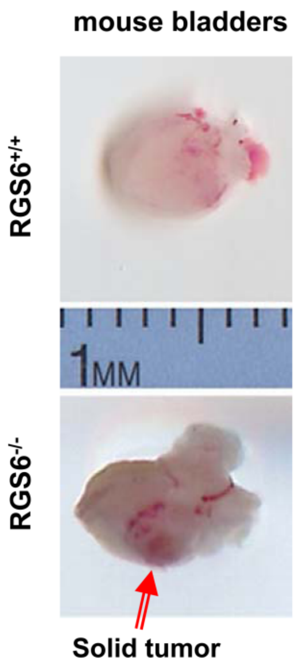

Fig S2. Representative image of BBN-induced solid tumor in RGS6$^{-/-}$ mouse bladder. Bladders were excised from RGS6$^{+/+}$ and RGS6$^{-/-}$ mice that were treated with BBN for 12 wks. Red arrow, solid tumor.

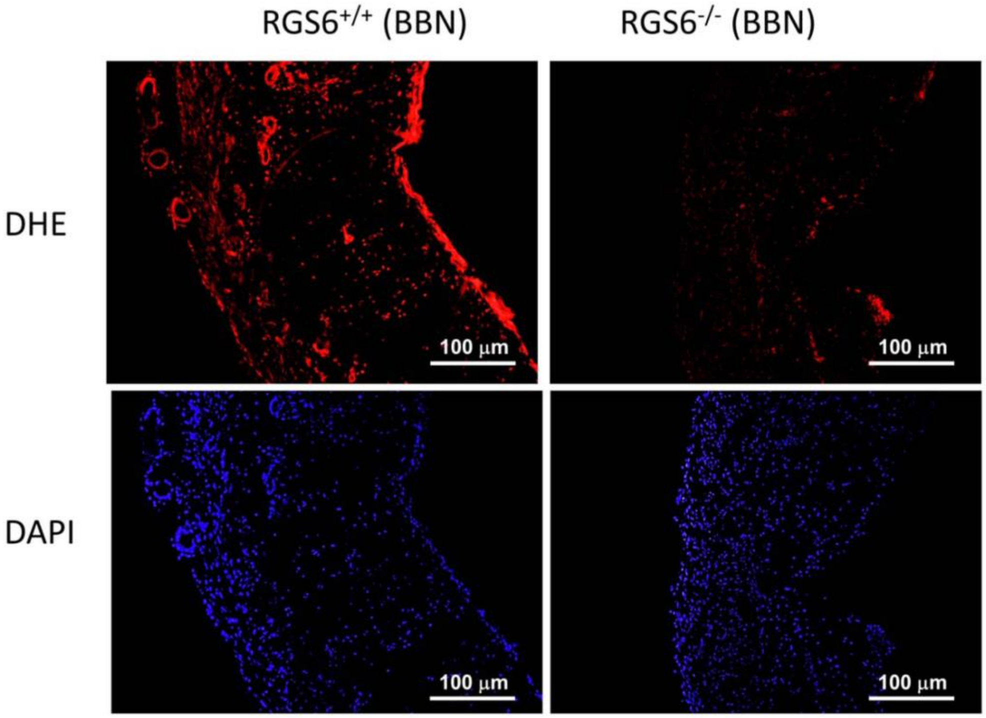

Fig S3. RGS6 loss is associated with a reduction in ROS generation within the mouse bladder. Mice were treated with 0.05% BBN water for 4 weeks. ROS level was measured using DHE staining.
